# Antiphospholipid Antibodies: Their Origin and Development

**DOI:** 10.3390/antib5020015

**Published:** 2016-06-02

**Authors:** Karl J. Lackner, Nadine Müller-Calleja

**Affiliations:** Institute of Clinical Chemistry and Laboratory Medicine, University Medical Center Mainz, D-55101 Mainz, Germany; nadine.prinz@unimedizin-mainz.de

**Keywords:** antiphospholipid antibodies, natural antibodies, innate immunity, B1 B cells

## Abstract

Antiphospholipid antibodies (aPL) are a hallmark of the antiphospholipid syndrome (APS), which is the most commonly acquired thrombophilia. To date there is consensus that aPL cause the clinical manifestations of this potentially devastating disorder. However, there is good evidence that not all aPL are pathogenic. For instance, aPL associated with syphilis show no association with the manifestations of APS. While there has been intensive research on the pathogenetic role of aPL, comparably little is known about the origin and development of aPL. This review will summarize the current knowledge and understanding of the origin and development of aPL derived from animal and human studies.

## 1. Introduction

Antibodies against phospholipids have been known for many decades as a hallmark of infection with *Treponema pallidum*. In 1906, Wassermann introduced a complement binding assay to detect antibodies in syphilitic patients [1]. Landsteiner soon hypothesized that the antigen might be a lipid rather than a protein [2], but it took over three decades until it was shown that the antigen in this assay was a phospholipid. This lipid was later called cardiolipin, because it was purified from myocardium [3]. With the continued use of cardiolipin based serologic assays for the diagnosis of syphilis it became apparent that a small group of patients with autoimmune disease, mostly systemic lupus erythematosus (SLE) had “false positive” tests caused by autoantibodies against cardiolipin. In the 1980s, researchers recognized that the presence of so called antiphospholipid antibodies (aPL) in SLE patients was associated with thromboembolic events and recurrent abortions, and the term anticardiolipin syndrome and later antiphospholipid syndrome (APS) was coined [4,5].

Today, there is broad consensus that aPL cause the clinical manifestations of APS. However, the underlying mechanisms are still a matter of controversy. This is perhaps related to the broad heterogeneity of aPL. Some aPL bind to anionic or neutral phospholipids coated to microtiter plates in the absence of proteins. Others can only bind in the presence of specific protein cofactors, e.g., β2-glycoprotein I (β2GPI) or prothrombin. The latter aPL are called cofactor dependent. Yet another group of aPL binds to the cofactors. These are also regarded as aPL even though their antigens in the strict sense are proteins or peptides. Some of the aPL detected by immunoassays also inhibit phospholipid dependent clotting assays. These are collectively called lupus anticoagulants (LA) [6]. It should be noted that there are some LA which do not react in the traditional immunoassays.

While there has been tremendous progress in the understanding of the pathogenic potential of aPL which has been reviewed repeatedly in the recent past [7,8,9,10,11,12], relatively little is known about the origin of aPL. As mentioned above patients suffering from syphilis develop antibodies against cardiolipin during their infection. However, these aPL do not induce the clinical symptoms of APS and must be regarded as different from pathogenic aPL. Similarly, it has been shown that other infectious diseases may cause the transient appearance of aPL. Again it appears that these transient aPL do not contribute to the development of APS. However, it has never been excluded that these transient antibodies might be pathogenic, but do not cause relevant damage, because of their transient nature. And finally, there have been reports of patients with monoclonal gammopathy with a monoclonal aPL. Interestingly, no such patient has been described with the clinical picture of APS. Until now there has been no scientifically proven explanation why some patients develop pathogenic aPL and subsequently APS. We will review the current knowledge about the origin and maturation of aPL and try to put forward some hypotheses on the development of pathogenic aPL.

## 2. Are aPL Part of the Natural Antibody Repertoire?

Natural antibodies appear without prior infection or immunization. The majority is of the immunoglobulin (Ig) M isotype, but IgG or IgA have also been observed [13,14]. They are secreted mainly by B1 cells, a specific subset of B-lymphocytes. Activation of B1 cells does not depend on antigenic challenge and T-cell help, but can be elicited by constituents of innate immunity, e.g., pathogen associated molecular patterns (PAMPs). Natural antibodies are usually of low to moderate affinity but cross-react with several related antigens including autoantigens. Sequence analysis shows that natural antibodies are usually very close to germline sequences with few if any somatic mutations. It is postulated that natural antibodies constitute a rapid, first line response to infection that bridges the time needed by adaptive immunity to develop specific antibodies. An example are antibodies to phosphorylcholine, a constituent of Gram positive cell walls. Lack of B1 cells severely compromises the resistance to bacterial infections. Interestingly, also antibodies against phosphatidylcholine have been identified which is a component of senescent cell membranes. This suggests that natural antibodies also play a role in the removal of dying cells. This function in removal of possibly antigenic debris might also explain the protection against autoimmunity conferred by B1 cells.

It has been proposed in the past that aPL belong to the natural antibodies [15,16], because they share many properties with these B1 cell derived antibodies. aPL tend to be polyspecific and there is overlap with other autoantibodies e.g., anti-DNA. Many aPL are germline encoded or exhibit only minor deviations from germline sequences (see below). However, final proof of this concept has not been provided. We will review the current evidence that aPL belong to the natural antibody repertoire and that even germline encoded aPL may be pathogenic.

### 2.1. Animal Models

Animal models permit a more detailed analysis of the mechanisms how aPL develop. Unfortunately, also in the mouse model, data are by no means conclusive. However, there is an interesting mouse model of APS which strongly supports the notion that aPL are natural antibodies. This model is based on immunization of animals with an aPL in the presence of an appropriate adjuvant. Mice immunized in this way develop their own aPL. This model has been described by the group of Yehuda Shoenfeld in the early 1990s and has been used by other researchers as a model of APS [17]. It was initially explained by the generation of anti-idiotypic antibodies. Immunization with an aPL was proposed to generate an antibody against this specific aPL. This was supposedly followed by generation of an anti-idiotype that would have similar binding specificity as the original aPL used for immunization [18]. This concept has never been proven experimentally. The time course of the antibody response makes this sequence of events highly unlikely. Pierangeli and colleagues [19] showed that immunized animals develop very rapidly, *i.e.*, within one week after the first immunization, their own aPL reactive against cardiolipin while no anti-β2GPI is induced in this time frame. Furthermore, most of these aPL are of the IgG and not the IgM isotype. Considering the antigen used for immunization, the time frame in which aPL occur, and the fact that most aPL produced in this model are of the IgG isotype, the usual response of the adaptive immune system to an antigenic challenge cannot account for this phenomenon. First, the antigen used for immunization has nothing to do with the immediate antibody response. Second, the adaptive immune system does not usually generate significant amounts of specific IgG antibodies within 1 week. Thus, it is highly likely that this immunization scheme somehow induces a natural antibody response. Apparently, most of the antibody produced is of the IgG-type which is unusual but clearly possible for natural antibodies. Most importantly, aPL induced by this protocol have been shown to be pathogenic *in vivo*. Immunized mice develop thrombophilia as well as pregnancy failure [17,20]. Thus, in summary we propose that this unique mouse model provides strong evidence that aPL of the IgG isotype belong to the natural antibody repertoire and that these aPL are pathogenic, at least in mice. Furthermore, the rapid induction of pathogenic aPL implies that antigen driven maturation is not an absolute requirement for pathogenicity.

Along these lines it may be relevant that aPL have been shown to sensitize antigen presenting cells including plasmacytoid dendritic cells (pDC) towards agonists of toll-like receptor (TLR) 7 and/or TLR8 [21]. As a consequence exposure to single stranded RNA (ssRNA) or other agonists leads to a massively increased secretion of type I interferon. Unbalanced activation of TLR7 in particular in pDC has been shown to induce autoimmunity and autoantibody production in mice [22,23,24]. Thus, the effects of aPL on pathways of innate immunity might help to better understand this mouse model of APS.

It should be noted that other induction schemes have been explored in mice and rabbits that also lead to pathogenic aPL. For instance, immunization of rabbits with lipid A can also rapidly induce pathogenic aPL [25].

### 2.2. Infections and aPL

In humans, many infectious diseases are associated with a transient or permanent rise of aPL of the IgM and IgG isotype. These include viral infections, e.g., parvovirus B-19, cytomegalovirus (CMV) and hepatitis C, as well as bacterial and parasitic infections, e.g., syphilis or helicobacter pylori infection [26]. Even though the production of specific aPL by the adaptive immune system cannot be ruled out and molecular mimicry is proposed as one possible underlying mechanism [27,28,29,30,31], the high frequency of a uniform antibody response to extremely different antigens should alert to the possibility of induction of natural antibodies. Another interesting aspect of the association of viral infections with aPL is the fact that there is a significant number of patients who develop thrombotic events [26,32,33,34]. While it is not proven that these events are caused by aPL, the undisputable coincidence raises the question whether these infection associated aPL may be pathogenic.

### 2.3. Analysis of Human Monoclonal aPL

Analysis of monoclonal aPL isolated from patients with APS or healthy individuals has provided important insights into the natural history of aPL. Several monoclonal aPL including aPL of the IgG isotype show a germline configuration as would be expected if they belong to the natural antibody repertoire. A thorough review of the available sequence data on human monoclonal aPL has been published [35]. Overall, the data obtained from sequence analysis of human monoclonal aPL provide a heterogeneous picture. Some aPL have a germline sequence, but many aPL clearly show all signs of antigen driven maturation. While this suggests that these antibodies are derived from typical adaptive immune responses, the presence of somatic mutations does not rule out that the original antibody was part of the natural repertoire and produced by B1cells. In fact, isotype switches and somatic mutations in B1 cell derived antibodies occurs and has been discussed as an escape mechanism of autoimmune disease [36].

Along these lines the group of Jean-Louis Pasquali and co-workers could show in a series of elegant experiments that B cell clones producing IgG aPL are present in APS patients as well as in healthy individuals [37,38]. These B cell clones were surprisingly heterogeneous in terms of V-region usage. Furthermore, this group confirmed the presence of aPL with germline configuration as well as aPL with somatic mutations. Their data suggest that low affinity aPL belong to the natural antibody repertoire and that by so far unknown triggers these aPL can undergo antigen driven maturation [39].

Thus, there is a large body of evidence that many aPL including aPL of the IgG isotype are natural antibodies. However, it is also clear that antigen driven maturation of aPL and in particular anti-β2GPI does occur. It is not known, if this occurs starting from the natural antibodies or from completely different B-cell clones.

### 2.4. What Is the Role of Antigen Driven Maturation?

As outlined above several groups have isolated IgG aPL with significant deviations from known germline sequences. Thus, antigen driven maturation of aPL has been unequivocally proven. Some investigators have put forward the hypothesis that antigen driven maturation is required to generate pathogenic aPL. Lieby and colleagues isolated an aPL with three somatic mutations from an APS patient. This antibody was pathogenic in an *in vivo* pregnancy model. When this antibody was modified back to the germline sequence it still bound to phospholipids but was no longer pathogenic [40]. The authors interpreted this finding as indicating that pathogenicity of aPL is induced by antigen driven maturation and that this process is perhaps a prerequisite of pathogenicity.

We have also isolated human monoclonal aPL with germline configuration and somatic mutations [41,42,43]. Binding specificity of two of these antibodies, RR7F which has a high homology to germline and HL5B which carries several somatic mutations, is similar. Both induce potentially pathogenic responses in monocytes and endothelial cells, but the required concentration of RR7F is approx. one order of magnitude higher than that of HL5B [21,44,45]. However, both monoclonal aPL induce thrombus formation in an *in vivo* model of venous thrombosis [46]. Our data support the concept that antigen driven maturation does increase the pathogenic potential of aPL but that it is not an indispensable prerequisite for pathogenicity. With respect to the requirements for pathogenicity of aPL data of Girardi and co-workers [47] are of relevance. They confirmed previous data that the human monoclonal aPL Mab519 is pathogenic in mice. It causes foetal resorption in pregnant mice. Mab519 was cloned from a healthy individual and deviates only non-significantly from germline sequence [48].

In summary, antigen driven maturation is not required for pathogenicity of aPL but apparently increases their pathogenic potential. It should be kept in mind though that neither class switch nor antigen driven mutations exclude a natural *i.e.*, B1 cell origin of aPL.

### 2.5. Memory B-cells

Few data are available on memory B-cells in APS. Again Lieby and co-workers have provided some insight into this issue by cloning antiphospholipid specific B-cells. They showed that in non-APS patients during acute episodes of Epstein-Barr virus (EBV) infection significant numbers of aPL-producing CD27 positive B-cells were detectable which the authors regarded as memory B-cells [36]. The origin of these cells in individuals who never had any manifestations of APS remains unclear. The presence of memory B-cells capable to secrete aPL is also supported by isolated cases of the development of APS after bone-marrow transplantation from an APS donor [49]. The presence of CD27 is not restricted to memory B cells but is also found in B1 cells [14]. It should be mentioned that there is an on-going scientific debate regarding the question if a distinct CD20^+^CD27^+^CD43^+^ positive B cell subset represents B1 cells [50,51,52]. Furthermore, the ability of B1 cells to mount a T-cell independent memory response is well established [53,54,55]. If CD27 identifies a B1 cell subset and B1 cells can confer long-lasting immunity, the data obtained by Lieby could also be interpreted as showing an increased number of a subset of B1 cells.

## 3. Genetic Aspects of aPL

Genetic predisposition to the development of aPL or even APS might provide additional clues regarding the origin of these antibodies. Unfortunately, available data are scarce. There are two genome wide associations studies (GWAS) available which address the genetic associations of aPL [56,57]. Both studies explicitly do not apply to APS but focus on the presence of aPL only. While no significant association of a genetic locus with anticardiolipin antibodies was detected, several potential loci associated with antibodies against β2GPI were identified. In particular, the apolipoprotien H (*APOH*) gene itself is associated with the presence of anti-β2GPI. This confirmed previous data from candidate gene approaches which had shown that certain polymorphisms of the *APOH* locus are associated with the presence of anti-β2GPI. Of particular relevance is rs4581 causing a missense mutation Val247Leu in domain V of β2GPI [58,59]. In our hands two other missense mutations were significantly associated with the presence of anti-β2GPI. These were rs52797880 (Ile122Val) and rs8178847 (Arg135His) in domain III of β2GPI [57]. It is not known yet, if one of these polymorphisms is causally related to the development of anti-β2GPI or if they are in linkage disequilibrium to the relevant polymorphism. Another locus associated with anti-β2GPI in both GWA studies was *MACROD2*. At present, no obvious explanation for this association has been found. And finally, similar to other autoimmune diseases several possible associations of aPL and APS with the human leukocyte antigen (HLA)-locus have been reported. This has been recently reviewed in detail [60]. There are two types of studies. The first analyses the association of major histocompatibility complex (MHC) genes with APS. The second analyses the association of MHC genes with aPL. In the latter most MHC associations were found with anti-β2GPI. This again suggests that anticardiolipin and anti-β2GPI may develop along different pathways.

The available data raise an intriguing question. Apparently, there is a strong association of anti-β2GPI with the *APOH* gene and possibly a few other genes including MHC genes while no genetic association of anticardiolipin antibodies has been described, in particular not with the *APOH* locus. This obvious genetic difference implies that the origin of these two aPL species might be different. There are two potential explanations: (1) anticardiolipin and anti-β2GPI develop completely independently from each other. This appears to be unlikely considering the high coincidence of both aPL; (2) Anti-β2GPI develop preferentially in persons who have also anticardiolipin. In this case, the *APOH* polymorphisms may affect the structure of β2GPI in a way which favours autoantibody formation against the protein. The crystal structure of β2GPI revealed that the protein consists of five domains which are arranged in a J-shaped elongated form much like beads on a string [61]. Later on it was shown that β2GPI can also attain a S-shaped and a circular form [62,63]. In fact, these are the conformations that β2GPI attains when it is not bound to phospholipids. In these two conformations an epitope comprising amino acids 40–43 in domain I is hidden within the tertiary structure of the protein. Transformation to the J-shaped conformation is required in order that specific anti-β2GPI can bind to this supposedly pathogenic epitope in domain I of the protein [64]. It is conceivable that missense mutations in β2GPI affect the accessibility of this epitope to the immune system or change the overall immunogenicity of β2GPI and thereby favour the development of anti-β2GPI. This scenario requires further scientific validation.

Regarding the relationship of anticardiolipin and anti-β2GPI, we made a relevant observation in a pair of human monoclonal aPL (HL5B and HL7G) isolated from the same patient [41,43]. Both aPL have a number of identical somatic mutations, but HL7G has some additional mutations indicating that it is more advanced by antigen driven maturation. While HL5B binds to cardiolipin in the absence of cofactors and does not bind to β2GPI, HL7G in addition binds to β2GPI. This observation shows that antigen driven maturation can transform anticardiolipin specific aPL to anti-β2GPI. We do not know if this occurs regularly and can be generalized, but our data show that this is one pathway to generate anti-β2GPI. In any case, it could explain the observation that anti-β2GPI is strongly associated to the *APOH* locus, while anticardiolipin is definitely not.

## 4. Conclusions and Outlook

**Figure 1:**
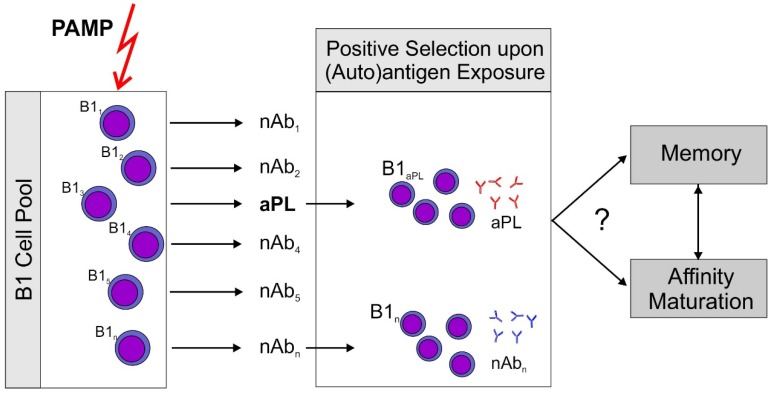
Proposed sequence of events leading to antiphospholipid antibodies (aPL). A non-specific stimulus by pathogen associated patterns (PAMP) which can activate pattern recognition receptors, e.g., toll-like receptors (TLR) stimulates an increase over basal antibody production by B1 cells. Subsequently, antigen producing B1 cell clones are positively selected by exposure to their (auto)antigen. This model could explain rapid aPL production induced by immunization of mice with an aPL. It should be noted that aPL themselves are able to sensitize immune cells to the action of ligands for TLR7, the receptor for single stranded RNA (ssRNA), by inducing TLR7 transcription and translocation to the endosome [21]. This could explain the role for aPL in this immunization scheme. Development of memory and antigen driven maturation have been described for B1 cells. However, there is only circumstantial evidence that this might occur with aPL producing clones.

We believe that the available data in the literature very strongly support the hypothesis that aPL are natural antibodies generated by B1 cells. Figure 1 depicts the basic concept which is at present only a working model and clearly needs substantial further experimental validation. There is ample evidence that aPL with germline sequence can be pathogenic even though it is likely that antigen driven maturation can increase the pathogenic potential of aPL. In particular, the development of aPL specific for β2GPI is very probably antigen driven. There is at least one documented case that an antibody against β2GPI evolved by somatic mutation from an anticardiolipin antibody. Since it has been shown in the past that B1 cells and the antibodies produced by them can undergo antigen driven maturation, antigen driven maturation does not argue against B1 cells being a major source of aPL. If aPL derive from B1 cells it can be expected that activation via innate immune processes rather than traditional HLA-dependent pathways of adaptive immunity may play a significant role in their development.

## Glossary

Germline sequence	Antibodies are encoded in the genome as every other protein. However, for certain segments (V, D, and J) of the variable chains of antibodies there are several coding gene segments. The term germline sequence refers to an antibody sequence encoded in the genome. Germline sequences can be modified by*→*antigen driven maturation. If an antibody has a germline encoded sequence this suggests that no antigen driven maturation has occurred, yet.
V,(D), J (or somatic) -recombination	The process of combining the gene segments for the desired V, D, and J-segments of the variable chains and of removal of surplus gene segments from the B cell genome is referred to as somatic recombination or V, (D), J-recombination. It is mediated by VDJ-recombinase, a multi-enzyme complex. Somatic recombination is the first step in antibody production that generates a huge potential diversity with more than 10^11^ theoretical combinations.
Somatic (hyper)mutation	During B-cell proliferation which occurs after antigen contact, the B-cell receptor locus can undergo an extremely high rate of somatic mutations which is several orders of magnitude greater than the spontaneous mutation rate. Most of the somatic mutations are found in specific regions of the antibody molecule, the so called hypervariable or complementarity determining regions (CDR). Somatic mutation generates B-cell clones which produce antibodies with different affinity to their antigen. The clones producing higher affinity antibodies are positively selected. Thus, somatic hypermutation is key to→antigen driven maturation of B-cell clones.
Antigen driven maturation	Also called affinity maturation, antigen driven maturation is the central process of adaptive immunity. By selecting higher affinity clones and deleting lower affinity clones, there is continuous improvement of antibody affinity to the relevant target antigen. A significant deviation of the sequence of an antibody from the known germline genes indicates antigen driven maturation.
Anti-idiotype	An idiotype describes the sum of the variable parts of a specific antibody. By this, it also includes the antigen binding site of the antibody. An anti-idiotype is an antibody that binds to a specific idiotype. In theory anti-idiotypes may mimick the antigen/epitope of the original antibody.

## Abbreviations

The following non-standard abbreviations are used in this manuscript:

aPL	antiphospholipid antibody
APS	antiphospholipid syndrome
β2GPI	β2 glycoprotein I
SLE	systemic lupus erythematosus

## References

[1] Wassermann A., Neisser A., Bruck C. (1906). Eine serodiagnostische Reaktion bei Syphilis. Dtsch. Med. Wochenschr..

[2] Landsteiner K., Müller R., Poetzl O. (1907). Zur Frage der Komplementbindungsreaktionen bei Syphilis. Wien. Klin. Wochenschr..

[3] Pangborn M. (1942). Isolation and purification of a serologically active phospholipid from beef heart. J. Biol. Chem..

[4] Harris E.N., Gharavi A.E., Boey M.L., Patel B.M., Mackworth-Young C.G., Loizou S., Hughes G.R. (1983). Anticardio-lipin antibodies: Detection by radioimmunoassay and association with thrombosis in systemic lupus erythematosus. Lancet.

[5] Hughes G.R. (1985). The anticardiolipin syndrome. Clin. Exp. Rheumatol..

[6] Bertolaccini M.L., Amengual O., Andreoli L., Atsumi T., Chighizola C.B., Forastiero R., de Groot P., Lakos G., Lambert M., Meroni P. (2014). 14th International Congress on Antiphospholipid Antibodies Task Force. Report on antiphospholipid syndrome laboratory diagnostics and trends. Autoimmun. Rev..

[7] Meroni P.L., Borghi M.O., Raschi E., Tedesco F. (2011). Pathogenesis of the antiphospholipid syndrome: understand-ding the antibodies. Nat. Rev. Rheumatol..

[8] Ioannou Y. (2012). The Michael Mason prize: Pathogenic antiphospholipid antibodies, stressed out antigens and the deployment of decoys. Rheumatology.

[9] Poulton K., Rahman A., Giles I. (2012). Examining how antiphospholipid antibodies activate intracellular signaling pathways: A systematic review. Sem. Arthritis. Rheum..

[10] Giannakopoulos B., Krilis S.A. (2013). The pathogenesis of the antiphospholipid syndrome. New Engl. J. Med..

[11] Du V.X., Kelchtermans H., de Groot P.G., de Laat B. (2013). From antibody to clinical phenotype, the black box of the antiphospholipid syndrome: Pathogenic mechanisms of the antiphospholipid syndrome. Thromb. Res..

[12] Merashli M., Noureldine J.H.A., Uthman I., Khamashta M. (2015). Antiphospholipid syndrome: An update. Eur. J. Clin. Investig..

[13] Panda S., Ding J.K. (2015). Natural antibodies bridge innate and adaptive immunity. J. Immunol..

[14] Rothstein T.L., Griffin D.O., Holodick N.E., Quach T.D., Kaku H. (2013). Human B-1 cells take the stage. Ann. New York Acad. Sci..

[15] Youinou P., Renaudineau Y. (2004). The antiphospholipid syndrome as a model for B cell-induced autoimmune diseases. Thromb. Res..

[16] Merrill J.T. (2006). Do antiphospholipid antibodies develop for a purpose?. Curr. Rheumatol. Rep..

[17] Bakimer R., Fishman P., Blank M., Sredni B., Djaldetti M., Shoenfeld Y. (1992). Induction of primary antiphospholipid syndrome in mice by immunization with a human monoclonal anticardiolipin antibody (H-3). J. Clin. Investig..

[18] Shoenfeld Y. (1994). Idiotypic induction of autoimmunity: A new aspect of the idiotypic network. FASEB J..

[19] Pierangeli S.S., Harris E.N. (1993). Induction of phospholipid-binding antibodies in mice and rabbits by immunization with human β2 glycoprotein 1 or anticardiolipin antibodies alone. Clin. Exp. Immunol..

[20] Pierangeli S.S., Liu S.W., Anderson G., Barker J.H., Harris E.N. (1996). Thrombogenic properties of murine anti-cardiolipin antibodies induced by beta 2 glycoprotein 1 and human immunoglobulin G antiphospholipid antibodies. Circulation.

[21] Prinz N., Clemens N., Strand D., Pütz I., Lorenz M., Daiber A., Stein P., Degreif A., Radsak M., Schild H. (2011). Antiphospholipid antibodies induce translocation of TLR7 and TLR8 to the endosome in human monocytes and plasmacytoid dendritic cells. Blood.

[22] Fukui R., Saitoh S., Kanno A., Onji M., Shibata T., Ito A., Onji M., Matsumoto M., Akira S., Yoshida N. (2011). Unc93B1 restricts systemic lethal inflammation by orchestrating Toll-like receptor 7 and 9 trafficking. Immunity.

[23] Yokogawa M., Takaishi M., Nakajima K., Kamijima R., Fujimoto C., Kataoka S., Terada Y., Sano S. (2014). Epicutaneous application of toll-like receptor 7 agonists leads to systemic autoimmunity in wild-type mice: A new model of systemic Lupus erythematosus. Arthritis Rheumatol..

[24] Takagi H., Arimura K., Uto T., Fukaya T., Nakamura T., Choijookhuu N., Hishikawa Y., Sato K. (2016). Plasmacytoid dendritic cells orchestrate TLR7-mediated innate and adaptive immunity for the initiation of autoimmune inflammation. Sci. Rep..

[25] Gotoh M., Matsuda J. (1996). Induction of anticardiolipin antibody and/or lupus anticoagulant in rabbits by immunization with lipoteichoic acid, lipopolysaccharide and lipid, A. Lupus.

[26] Abdel-Wahab N., Lopez-Olivo M.A., Pinto-Patarroyo G.P., Suarez-Almazor M.E. (2016). Systematic review of case reports of antiphospholipid syndrome following infection. Lupus.

[27] Gharavi A.E., Pierangeli S.S., Espinola R.G., Liu X., Colden-Stanfield M., Harris E.N. (2002). Antiphospholipid antibodies induced in mice by immunization with a cytomegalovirus-derived peptide cause thrombosis and activation of endothelial cells *in vivo*. Arthritis Rheum..

[28] Blank M., Krause I., Fridkin M., Keller N., Kopolovic J., Goldberg I., Tobar A., Shoenfeld Y. (2002). Bacterial induction of autoantibodies to beta2-glycoprotein-I accounts for the infectious etiology of antiphospholipid syndrome. J. Clin. Investig..

[29] Gharavi A.E., Pierangeli S.S., Harris E.N. (2003). Viral origin of antiphospholipid antibodies: Endothelial cell activa-tion and thrombus enhancement by CMV peptide-induced APL antibodies. Immunobiology.

[30] Shoenfeld Y., Blank M., Cervera R., Font J., Raschi E., Meroni P.L. (2006). Infectious origin of the antiphospholipid syndrome. Ann. Rheum. Dis..

[31] Martin E., Winn R., Nugent K. (2011). Catastrophic antiphospholipid syndrome in a community-acquired methicillin-resistant Staphylococcus aureus infection: A review of pathogenesis with a case for molecular mimicry. Autoimmun. Rev..

[32] Justo D., Finn T., Atzmony L., Guy N., Steinvil A. (2011). Thrombosis associated with acute cytomegalo-virus infection: A meta-analysis. Eur. J. Intern. Med..

[33] Uthman I., Tabbarah Z., Gharavi A.E. (1999). Hughes syndrome associated with cytomegalovirus infection. Lupus.

[34] Nakayama T., Akahoshi M., Irino K., Kimoto Y., Arinobu Y., Niiro H., Tsukamoto H., Horiuchi T., Akashi K. (2014). Transient antiphospholipid syndrome associated with primary cytomegalovirus infection: A case report and literature review. Case Rep. Rheumatol..

[35] Giles I.P., Haley J.D., Nagl S., Isenberg D.A., Latchman D.S., Rahman A. (2003). A systematic analysis of sequences of human antiphospholipid and anti-b2-glycoprotein I antibodies: The importance of somatic mutations and certain sequence motifs. Semin. Arthritis Rheum..

[36] Elkon K., Casali P. (2008). Nature and functions of autoantibodies. Nat. Clin. Pract. Rheumatol..

[37] Lieby P., Soley A., Levallois H., Hugel B., Freyssinet J.-M., Cerutti M., Pasquali J.-L., Martin T. (2001). The clonal analysis of anticardiolipin antibodies in a single patient with primary antiphospholipid syndrome reveals extreme antibody heterogeneity. Blood.

[38] Lieby P., Soley A., Knapp A.-M., Cerutti M., Freyssinet J.-M., Pasqualit J.-L., Martin T. (2003). Memory B cells producing somatically mutated antiphospholipid antibodies are present in healthy individuals. Blood.

[39] Pasquali J.-L., Nehme H., Korganow A.-S., Martin T. (2004). Antiphospholipid antibodies: Recent progresses on their origin and pathogenicity. Joint Bone Spine.

[40] Lieby P., Poindron V., Roussi S., Klein C., Knapp A.M., Garaud J.C., Cerutti M., Martin T., Pasquali J.L. (2004). Patho-genic antiphospholipid antibody: An antigen-selected needle in a haystack. Blood.

[41] von Landenberg C., Lackner K.J., von Landenberg P., Lang B., Schmitz G. (1999). Isolation and character-rization of two human monoclonal antiphospholipid IgG from patients with autoimmune disease. J. Autoimmun..

[42] Buschmann C., Fischer C., Ochsenhirt V., Neukirch C., Lackner K.J., von Landenberg P. (2005). Generation and characterization of three monoclonal IgM antiphospholipid antibodies recognizing different phospholipid antigens. Ann. N. Y. Acad. Sci..

[43] Prinz N., Häuser F., Lorenz M., Lackner K.J., von Landenberg P. (2011). Structural and functional characterization of a human IgG monoclonal antiphospholipid antibody. Immunobiology.

[44] Prinz N., Clemens N., Canisius A., Lackner K.J. (2013). Endosomal NADPH-oxidase is critical for induction of the tissue factor gene in monocytes and endothelial cells. Lessons from the antiphospholipid syndrome. Thromb. Haemost..

[45] Müller-Calleja N., Köhler A., Siebald B., Canisius A., Orning C., Radsak M., Stein P., Mönnikes R., Lackner K.J. (2015). Cofactor-independent antiphospholipid antibodies activate the NLRP3-inflammasome via endosomal NADPH-oxidase: Implications for the antiphospholipid syndrome. Thromb. Haemost..

[46] Manukyan D., Müller-Calleja N., Jäckel S., Luchmann K., Mönnikes R., Kiouptsi K., Reinhardt C., Jurk K., Walter U., Lackner K.J. (2016). Cofactor Independent Human Antiphospholipid Antibodies Induce Venous Thrombosis in Mice. J. Thromb. Haemost..

[47] Girardi G., Berman J., Redecha P., Spruce L., Thurman J.M., Kraus D., Hollmann T.J., Casali P., Caroll M.C., Wetsel R.A. (2003). Complement C5a receptors and neutrophils mediate fetal injury in the antiphospholipid syndrome. J. Clin. Investig..

[48] Ikematsu W., Luan F.L., La Rosa L., Beltrami B., Nicoletti F., Buyon J.P., Meroni P.L., Balestrieri G., Casali P. (1998). Human anticardiolipin monoclonal autoantibodies cause placental necrosis and fetal loss in BALB/c mice. Arthritis Rheum..

[49] Ritchie D.S., Sainani A., D'Souza A., Grigg A.P. (2005). Passive donor-to-recipient transfer of antiphospholipid syndrome following allogeneic stem-cell transplantation. Am. J. Hematol..

[50] Griffin D.O., Holodick N.E., Rothstein T.L. (2011). Human B1 cells in umbilical cord and adult peripheral blood express the novel phenotype CD20^+^CD27^+^CD43^+^CD70^−^. J. Exp. Med..

[51] Tangye S.G. (2013). To B1 or not to B1: That really is still the question!. Blood.

[52] Inui M., Hirota S., Hirano K., Fujii H., Sugahara-Tobinai A., Ishii T., Harigae H., Takai T. (2015). Human CD43^+^ B cells are closely related not only to memory B cells phenotypically but also to plasmablasts developmentally in healthy individuals. Int. Immunol..

[53] Allugupalli K.R., Leong J.M., Woodland R.T., Muramatsu M., Honjo T., Gerstein R.M. (2004). B1b lymphocytes confer T cell-independent long-lasting immunity. Immunity.

[54] Yang Y., Ghosn E.E., Cole L.E., Obukhanych T.V., Sadate-Ngatchou P., Vogel S.N., Herzenberg L.A., Herzenberg L.A. (2012). Antigen-specific memory in B-1a and its relationship to natural immunity. Proc. Natl. Acad. Sci. USA.

[55] Yang Y., Ghosn E.E., Cole L.E., Obukhanych T.V., Sadate-Ngatchou P., Vogel S.N., Herzenberg L.A., Herzenberg L.A. (2012). Antigen-specific antibody responses in B-1a and their relationship to natural immunity. Proc. Natl. Acad. Sci. USA.

[56] Kamboh M.I., Wang X., Kao A.H., Barmada M.M., Clarke A., Ramsey-Goldman R., Manzi S., Demirci F.Y. (2013). Genome-wide association study of antiphospholipid antibodies. Autoimmun. Dis..

[57] Müller-Calleja N., Rossmann H., Müller C., Wild P., Blankenberg S., Pfeiffer N., Binder H., Beutel M.E., Manukyan D., Zeller T. (2016). Antiphospholipid antibodies in a large population-based cohort: genome-wide associations and effects on monocyte gene expression. Thromb. Haemost..

[58] Hirose N., Williams R., Alberts A.R., Furie R.A., Chartash E.K., Jain R.I., Sison C., Lahita R.G., Merrill J.T., Cucurull E. (1999). A role for the polymorphism at position 247 of the beta2-glyco-protein I gene in the generation of anti-beta2-glycoprotein I antibodies in the antiphospholipid syndrome. Arthritis Rheum..

[59] Chamorro A.J., Marcos M., Mirón-Canelo J.A., Cervera R., Eapinosa G. (2012). Val247Leu β2-glycoprotein-I allelic variant is associated with antiphospholipid syndrome: Systematic review and meta-analysis. Autoimmun. Rev..

[60] Sebastiani G.D., Iuliano A., Cantarini L., Galeazzi M. (2016). Genetic aspects of the antiphospholipid syndrome: An update. Autoimmun. Rev..

[61] Schwarzenbacher R., Zeth K., Diederichs K., Gries A., Kostner G.M., Laggner P., Prassl R. (1999). Crystal structure of human beta2-glycoprotein I: Implications for phospholipid binding and the antiphospholipid syndrome. EMBO J..

[62] Hammel M., Kriechbaum M., Gries A., Kostner G.M., Laggner P., Prassl R. (2002). Solution structure of human and bovine beta(2)-glycoprotein I revealed by small-angle X-ray scattering. J. Mol. Biol..

[63] Agar C., van Os G.M., Mörgelin M., Sprenger R.R., Marquart J.A., Urbanus R.T., Derksen R.H., Meijers J.C., de Groot P.G. (2010). Beta2-glycoprotein I can exist in 2 conformations: Implications for our understanding of the antiphospholipid syndrome. Blood.

[64] Ninivaggi M., Kelchtermans H., Lindhout T., de Laat B. (2012). Conformation of beta2glycoprotein I and its effect on coagulation. Thromb. Res..

